# Cross-Talk in Viral Defense Signaling in Plants

**DOI:** 10.3389/fmicb.2016.02068

**Published:** 2016-12-21

**Authors:** Ju Y. Moon, Jeong M. Park

**Affiliations:** ^1^Plant Systems Engineering Research Center, Korea Research Institute of Bioscience and BiotechnologyDaejeon, South Korea; ^2^Department of Biosystems and Bioengineering, University of Science and TechnologyDaejeon, South Korea

**Keywords:** defense responses, *R* gene, RNAi, viral suppressor of RNAi, virus

## Abstract

Viruses are obligate intracellular parasites that have small genomes with limited coding capacity; therefore, they extensively use host intracellular machinery for their replication and infection in host cells. In recent years, it was elucidated that plants have evolved intricate defense mechanisms to prevent or limit damage from such pathogens. Plants employ two major strategies to counteract virus infections: resistance (*R*) gene-mediated and RNA silencing-based defenses. In this review, plant defenses and viral counter defenses are described, as are recent studies examining the cross-talk between different plant defense mechanisms.

## Introduction

Plant viruses comprise an important group of pathogens causing a range of plant diseases that are often responsible for significant losses in crop production. Among the wide range of known plant viruses, most viruses have a very limited host range and only a few viruses cause severe disease symptoms (Dawson and Hilf, 1992). Even though viruses contain relatively simple genomes, the molecular basis of the mechanisms by which plant viruses infect their hosts and the signaling components involved in host resistance are not well defined.

The immune response against bacterial or fungal pathogens often relies on recognition of the conserved molecules associated with a group of pathogens, designated pathogen-associated molecular patterns (PAMPs), by pattern recognition receptors (PRRs) (Boller and He, 2009). Upon PAMP recognition, activated PRRs induce PAMP-triggered immunity (PTI) (Monaghan and Zipfel, 2012). PTI against viral pathogens has been primarily described in mammalian cells, but not in plant cells (Calil and Fontes, 2016). However, several recent studies provided evidence that PTI and related components are also involved in antiviral defense responses in plants (Korner et al., 2013; Nicaise, 2014; Iriti and Varoni, 2015; Calil and Fontes, 2016; Nicaise and Candresse, 2016; Niehl et al., 2016). In general, plants defense responses triggered against viral pathogens are based on RNA- or protein-mediated resistance. The RNA-mediated resistance response is a basal defense response to viral invasion that mainly involves the RNA silencing pathway of the host, which mediates the cleavage of viral RNA. Compared to this basal defense response, the host resistance (R) protein-mediated defense response against viral pathogens is far more robust, in most cases limiting viral replication and spread to inoculated leaves (Zhou and Chai, 2008; Verlaan et al., 2013; Nakahara and Masuta, 2014). In this review, we summarize molecular mechanisms underlying two major defense pathways employed during plant resistance to viral pathogens and highlight a few studies addressing the cross-talk between these defense pathways.

## RNA Silencing in Viral Defense

RNA gene silencing, also termed RNA interference (RNAi), which acts as a basal defense mechanism against viruses, is one of the main plant immune responses against viral pathogens (Vaucheret, 2006; Ding and Voinnet, 2007). Most viruses that cause disease in plants have RNA genomes containing imperfect regulatory stem-loops, which are copied into complementary double-stranded RNA (dsRNA) replication intermediates by virus-encoded RNA-dependent RNA polymerases (RDRs) (Ruiz-Ferrer and Voinnet, 2009). The dsRNAs are then recognized by a host ribonuclease III-like protein, namely, Dicer-like (DCL), and then processed into 21–24-nucleotide short interfering RNAs (siRNAs). The siRNAs are recruited to a functional RNA-induced silencing complex (RISC) and then act as guides to direct RISC to their target viral RNA molecules, which have complementary sequences (Ruiz-Ferrer and Voinnet, 2009). Consequently, viral RNAs are degraded by the core components of RISC, which are members of the Argonaut (AGO) protein family (Vaucheret, 2008). The antiviral RNAi response is effective in various species (Katiyar-Agarwal and Jin, 2010), even though it is slow and thus viral infections are often not completely cleared.

The concept of PTI against viral pathogens is currently confined to animals because receptors that sense RNA or DNA viruses as ligands have only been identified in animals (Takeuchi and Akira, 2009). In plants, dsRNAs produced during virus infection are also regarded as viral PAMPs (Ding, 2010; Jensen and Thomsen, 2012). The RNA silencing pathway was assumed to play a role in the immune responses that recognize such dsRNAs in plants, unlike in animals (Ding and Voinnet, 2007). However, a few recent publications indicate that the known PTI components are involved in dsRNA recognition and that the reaction is an immune response distinct from the RNA silencing pathway. Therefore, these studies claim that PTI against viral pathogens is preserved in plants and animals (Korner et al., 2013; Nicaise, 2014; Nicaise and Candresse, 2016; Niehl et al., 2016). However, there is no direct evidence to explain how dsRNAs are recognized in plants; therefore, further studies are needed to determine whether an animal-like mechanism underlies dsRNA-mediated PTI in plants.

**FIGURE 1:**
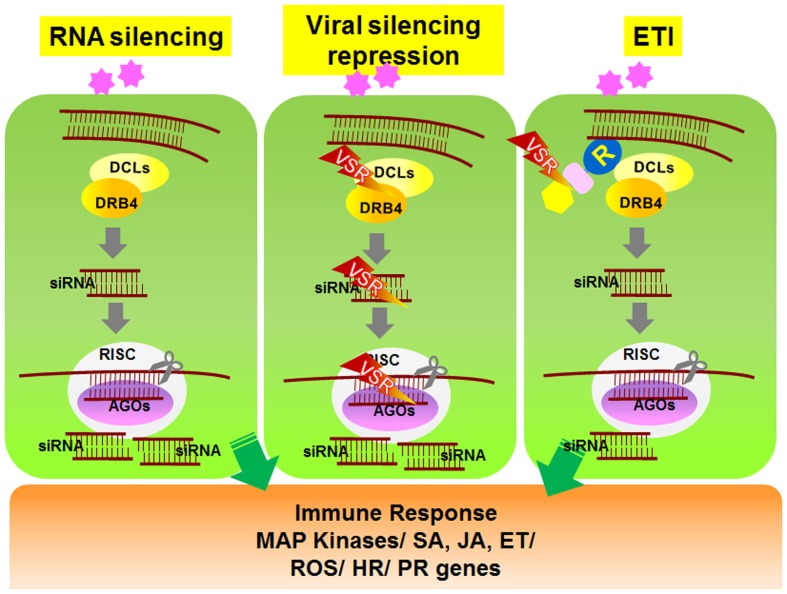
**Schematic model of RNA silencing- and *R*-mediated responses in plant cells**. Upon amplification of viruses in plant cells, viral double-stranded RNAs (dsRNAs) activate RNA silencing mechanisms. Viral dsRNAs are processed into small RNA fragments (siRNAs) by DCL1 and its cofactor DRB4. The siRNAs are recruited to RISC, which is associated with AGO protein. RISC/AGO/siRNA then targets and degrades complementary viral transcripts (left panel). Viruses express genes encoding VSR proteins that inhibit the regulation and activation of gene silencing mechanisms (center panel). In response, several R proteins recognize the VSRs and induce downstream ETI responses (right panel). DCL, Dicer-like; DRB, dsRNA-binding protein; siRNA, small interfering RNA; AGO, Argonaute; RISC, RNA-induced silencing complex; VSR, viral suppressors of RNA silencing.

To overcome RNAi-mediated host defense, plant viruses frequently encode viral suppressors of RNAi (VSRs) that perturb the plant RNA silencing pathway (Ding and Voinnet, 2007). VSRs have been isolated from nearly all plant virus families. In addition to suppressing RNAi silencing during viral pathogenesis, most VSRs identified to date play important roles in replication, assembly, or movement of viruses. Although the primary sequences and structures of these VSR proteins vary considerably, most VSR-mediated suppression is thought to occur via two general mechanisms (**Figure 1**). Some VSRs, such as potyviral HcPro, *Beet Yellow Virus* P21 protein, *Peanut Clump Virus* P15 protein, and TCV coat protein (CP or P38), sequester small RNA duplexes by binding to short or long viral dsRNAs, which then leads to impaired assembly of AGOs into RISCs (Lakatos et al., 2006; Carbonell and Carrington, 2015). Alternatively, some VSRs impede the activity of AGO proteins that have a central role in the anti-viral RNA silencing pathway (Carbonell and Carrington, 2015). For example, *Cucumber Mosaic Virus* 2b protein suppresses RISC activity through a physical interaction with the PAZ domain of AGO1 (Duan et al., 2012). Similarly, two other viral VSR proteins, *Sweet Potato Mild Mottle Virus* (SMMV) P1 and TCV CP, also directly interact with AGO proteins through glycine/tryptophan (GW/WG) repeat motifs, which resemble the AGO1-binding peptides on RISC (Azevedo et al., 2010; Giner et al., 2010). These findings demonstrate that VSR suppression of RNAi silencing might involve independently evolved VSR proteins that show functional overlap (Carbonell and Carrington, 2015). Studies on VSRs will not only improve our understanding of plant–virus interactions, but they will also help elucidate the signaling mechanism underlying host RNA silencing pathways.

## Resistance Gene-Mediated Defense Responses Against Viral Pathogens

To circumvent PTI, pathogens produce effectors that suppress immune responses triggered by active PRRs (Deslandes and Rivas, 2012). The bacterial pathogens usually encode ∼20–30 highly regulated effectors that are secreted directly into the host cytoplasm. Although individual effectors from closely related bacterial strains exhibit functional diversity, they possess highly redundant activities and extensive interchangeability (Cunnac et al., 2009; Deslandes and Rivas, 2012). This also applies to viral proteins such as movement proteins (MPs), and replicase proteins, which act as avirulent (Avr) factors (Kachroo et al., 2006).

Resistance (*R*) genes have evolved in plants as a countermeasure to the effect of pathogen effectors on PTI (Jones and Dangl, 2006). *R* genes mediate effector-triggered immunity (ETI), which is a highly amplified version of PTI (Jones and Dangl, 2006). Many *R* genes have been identified, which confer resistance to diverse pathogens including bacteria, fungi, oomycetes, insects, and viruses (Jones and Dangl, 2006; Kachroo et al., 2006). Notable examples of *R* genes conferring resistance against viral pathogens include tobacco “N” against TMV, “Rx1/2” in potato against Potato Virus X (PVX), and “HRT” and “RCY1” against TCV and CMV in *Arabidopsis*, respectively (Whitham et al., 1994; Bendahmane et al., 1999, 2000; Cooley et al., 2000; Takahashi et al., 2001). The *R* genes are largely dominant, whereas some genes exhibit recessive, tolerance, or partial resistance characteristics. Moreover, dominant R genes *HRT* and *RCY1* require recessive factors to confer resistance (Cooley et al., 2000; Takahashi et al., 2001). Since viruses require host factors for their infection (termed susceptibility factors), loss of these can also confer resistance to viral pathogens. Such resistance is often recessive (Truniger and Aranda, 2009). Notably, most such recessive *R* genes have been analyzed in potyviruses and encode translation initiation factors of the 4E or 4G family (eIF4E/eIF4G) (Kang et al., 2005; Truniger and Aranda, 2009). Interestingly, EF1A is required for Soybean Mosaic virus (SMV)-induced endoplasmic reticulum (ER) stress and, therefore, replication of SMV (Luan et al., 2016). Consequently, silencing of *EF1A* inhibits SMV replication and confers resistance against SMV.

The majority of dominant *R* proteins contain nucleotide-binding site (NBS) and leucine rich repeat (LRR) domains (Collier and Moffett, 2009), which is also the case for *R* genes that confer resistance against viral pathogens (de Ronde et al., 2014). The NBS-LRR R proteins can be further subcategorized as putative coiled-coil- or toll-interleukin-1 receptor-like (TIR)-type proteins based on the presence of these domains at their N-termini (Collier and Moffett, 2009). TIR, NBS, and LRR domains are also found in *Drosophila* and human receptor proteins involved in innate immunity (Nürnberger et al., 2004), suggesting that the animal and plant proteins evolutionarily diverged from a common ancestor and that and that similar modules were selected to regulate innate immune responses.

While only selected R proteins show direct interactions with Avr factors (Dodds et al., 2006; Ueda et al., 2006; Cesari et al., 2013), most R proteins are thought to act indirectly via other intermediary host proteins. This is further explained by the “guard/decoy” model, which describes how R proteins guard host accessory proteins (guardees), and pathogen effector-mediated alteration of the guardees results in the activation of R protein (Jones and Dangl, 2006; Collier and Moffett, 2009; Dodds and Rathjen, 2010). For example, N protein from tobacco indirectly recognizes a p50 helicase fragment of the TMV replicase protein via a chloroplast-localized N receptor-interacting protein 1 (NRIP1) (Caplan et al., 2008). Upon TMV infection, NRIP1 residing in the chloroplast translocates to the cytoplasm and nucleus. Cytosolic NRIP1 associates with TMV replicase and then recruits N protein through a direct interaction between NRIP1 and the TIR domain of N (Ueda et al., 2006).

Unlike viral pathogens, both fungal and bacterial genomes encode multiple Avr factors, which are thought to play a role in the suppression of PTI. However, viruses appear to compensate for the presence of a single Avr effector by undergoing frequent alterations in the critical amino acid sequences without drastically changing the protein structure. Host R protein-mediated recognition of the modified Avr factor then depends on the relative affinity between R protein and the modified Avr factor. For instance, several hypervirulent strains of TCV isolated from *in planta*-propagated TCV are able to escape HRT-mediated recognition and cause disease in resistant plants (Wobbe et al., 1998; Zhu et al., 2013).

## Cross-Talk Between RNAi- and *R* Gene-Mediated Anti-Viral Defense Responses

**FIGURE 2:**
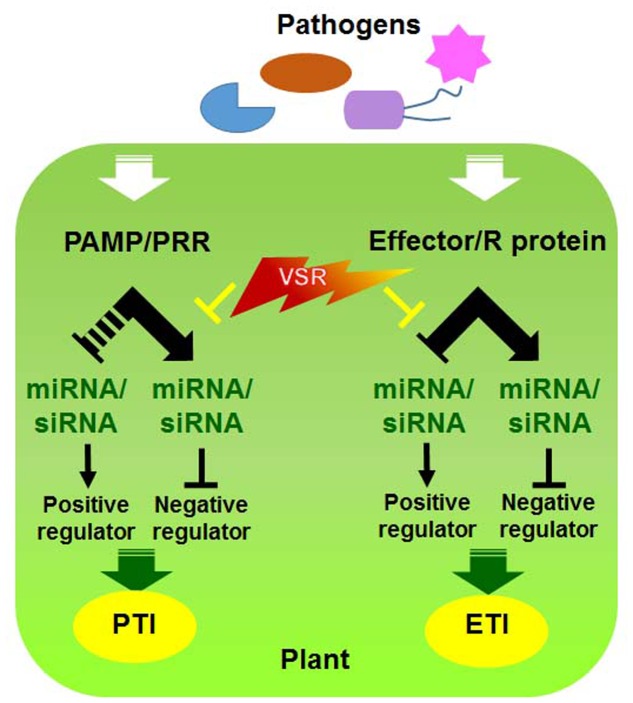
**Interactions between viral silencing suppression and host factors involved in PTI and ETI**. Model of molecular virus–host interactions in RNA silencing and PRR/R-mediated resistance [modified from (Katiyar-Agarwal and Jin, 2010)].

Since both RNAi and *R* gene-mediated pathways participate in antiviral defense, it is plausible that these pathways undergo cross-talk to maximize the efficiency of defense responses against viral infections (Nakahara and Masuta, 2014). Indeed, viral pathogens often encode a single protein that functions as a suppressor of RNAi as well as an Avr effector (**Figure 2**) (Palanichelvam et al., 2000; Eggenberger et al., 2008; Katiyar-Agarwal and Jin, 2010; Wen et al., 2012; Zhu et al., 2013). For example, TMV replicase and TCV CP function as VSRs and are recognized by N and HRT, respectively, to induce the HR (Wang et al., 2012; Zhu et al., 2013). However, it is currently unclear how they communicate with each other and whether they assist each other to increase disease resistance or have sequential defense functions and thereby act individually.

Recently, a few studies provided molecular evidence that these two defense mechanisms are associated with each other (Li et al., 2012; Shivaprasad et al., 2012; Verlaan et al., 2013; Zhu et al., 2013). Several components involved in host RNA silencing mechanisms have recently been shown to be required for *R* gene-mediated defense. For example, double-stranded RNA binding protein (DRB) four interacts with HRT and is required for HRT stability (Zhu et al., 2013). In addition, *R* genes against Tomato yellow leaf curl virus were recently shown to encode DFDGD-class RDR (Verlaan et al., 2013). Interestingly, activation of HRT-mediated resistance is not dependent on the RNA silencing suppressor activity of CP and is not associated with the accumulation of TCV-specific small RNA. This finding suggests that the HRT-mediated signaling pathway recruits components of the RNA silencing pathway, but this resistance response is not associated with the cleavage of viral RNA.

It is likely that alteration of small RNAs derived from viral infections plays a role in regulating *R* gene expression levels, thereby regulating resistance signaling (Li et al., 2012; Shivaprasad et al., 2012), rather than direct regulation by VSR activity. For instance, Li et al. (2012) observed that *miR6019* and *miR6020* in tobacco cause specific cleavage of transcripts of *N* and its homologs by binding to the complementary sequence of a conserved region encoding the TIR domain of the N protein. Furthermore, phasiRNA synthesis from the *N* coding sequence via overexpression of *miR6019* was accompanied by reductions in *N* transcript levels and *N*-mediated resistance against TMV (Li et al., 2012). In addition, a group of 22 nt miRNAs from the *miR482/2118* superfamily targets numerous NLRs within *Solanaceae* species. These miRNAs target highly conserved sequences in the genes encoding predicted NLR proteins (Zhai et al., 2011; Shivaprasad et al., 2012). Activation of VSR induces quantitative changes of whole small RNA species in host cells. Interestingly, VSRs upregulate the transcript levels of the targeted NLRs by attenuating the production or activity of *miR482/2118* family members. The *miR482/2118* family members are thought to ordinarily down-regulate their target *NLR* genes but upregulate these genes only when they are required for plant resistance via the VSRs of viral pathogens (Padmanabhan and Dinesh-Kumar, 2014). Altogether, these studies suggest that the RNA silencing response is integrated with *R* gene-mediated anti-viral resistance responses; however, it is not yet clear whether degradation of the viral genome via host RNA silencing-mediated defense is necessary for *R* gene-mediated defense.

## Concluding Remarks

Since the zig-zag model was first proposed by Jones and Dangl (2006), many interactions between plant and bacterial pathogens have been reported, in which a pathogen suppresses or alters PTI by effectors, and plants have developed induced ETI, a stronger type of defense against effectors, during evolution (Boller and He, 2009). Long-term plant disease resistance studies of viral pathogens have revealed RNA silencing and *R* gene-mediated defense responses. In recent years, studies of the relationship between these two resistance responses have enhanced understanding of the interaction between plants and viruses. As genome analysis techniques are developed, understanding of plant–virus interactions increases. Kontra et al. (2016) recently reported that the tombusviral P19 suppressor preferentially affects virus-derived small interfering RNAs rather than endogenous host miRNAs in virus-infected plants. The authors suggested that the relationship between VSRs and host RNA silencing, as well as their contribution to the virulence of viruses, should be reconsidered. In parallel, Li et al. (2013) revealed a role for miRNAs in translational inhibition as well as silencing in plants and demonstrated that this process occurs in the ER. It would be interesting to integrate our knowledge of the roles of the ER in viral pathogenesis and in *R* gene-mediated defense responses (Jheng et al., 2014; Verchot, 2014; Moon et al., 2016). Uncovering the subcellular localization of small RNAs, VSR, and R protein will be critical for understanding how the two antiviral pathways interact. Although the concept of PTI and ETI is less clear in viral pathogenesis than in bacterial pathogenesis at present, future in-depth studies of the two anti-viral defenses and cross-talk between them will enhance understanding of plant immune responses, as well as to bacteria and fungi.

## Author Contributions

JM wrote the paper. JP wrote and edited the paper.

## Conflict of Interest Statement

The authors declare that the research was conducted in the absence of any commercial or financial relationships that could be construed as a potential conflict of interest.
